# Factors Influencing User Satisfaction in Accessing Health Data: Cross-Sectional Survey of United Kingdom Adults

**DOI:** 10.2196/75935

**Published:** 2026-04-06

**Authors:** Maria Xenou, Omar Al-Ubaydli

**Affiliations:** 1 UCL Institute of Health Informatics, University College London, 222 Euston Rd, London, London, NW1 2DA, United Kingdom, 44 2035495969; 2 Department of Economics and Mercatus Center, George Mason University, 4400 University DriveFairfax, VA, 22030, United States

**Keywords:** patient health record, user satisfaction, national survey, digital health, United Kingdom

## Abstract

**Background:**

While patient health records (PHRs) are becoming ubiquitous, nationwide evidence on the drivers of user satisfaction in the United Kingdom remains scarce.

**Objective:**

This study aimed to quantify determinants of PHR user satisfaction in a nationally representative sample of United Kingdom adults and to contrast real-world experiences with hypothetical expectations among individuals without PHR exposure.

**Methods:**

We conducted a cross-sectional online survey in March 2022 using stratified quota sampling (eg, age, sex, ethnicity) through the Prolific platform. Of 1001 respondents, 533 (53%) were female (mean age 41, SD13) years, 468 (47%) reported previous PHR use (“experienced” cohort), and 533 (53%) respondents did not (“hypothetical” cohort). Primary outcomes were five satisfaction items (5-point Likert), overall PHR evaluation (0‐100), and stated/anticipated PHR functions. Two-sample *t*-tests with unequal variances examined between-group differences; multivariate analysis of variance (MANOVA) assessed demographic equivalence. Significance was set at *P*<.05.

**Results:**

Respondents with PHR experience rated their record easy-to-use in 79% (370/468) of cases versus an anticipated 93% (498/533) among nonusers (*P*<.001). Experienced users rated appointment-scheduling time-saving at 68% (319/468) compared with 72% (387/533) of nonusers (*P*=.17). Mean overall evaluation scores were 71.0 (SD 19.5) versus 74.2 (SD 18.6) (t₉₉₇=2.25, *P*=.02). MANOVA confirmed demographic balance (Wilks Λ=.99, F₇,₉₉₃=1.48, *P*=.18). Preferred functions across cohorts were viewing personal health information (experience 81%, hypothetical 90%) and lab results (50% vs 76%). Users lacking experience consistently over-estimated future use of carer-related functions (eg, children’s data: 36% vs 12%, *P*<.001).

**Conclusions:**

Adults in the United Kingdom value PHRs primarily for personal data access and scheduling convenience. Nonusers displayed optimistic expectations about carer-related features and breadth of functionality, indicating an information gap. Enhancing transparency about current capabilities and expanding features that facilitate caregiving could increase satisfaction and adoption.

## Introduction

### Background

Electronic access to health information is integral to patient-centered care [1]. In the United Kingdom, 73% of adults go online first for health information [2], and the National Health Service (NHS) aims to universalize digital data access under the ethos of “no decision about me without me” [3]. People may view clinical data through provider-tethered electronic medical records (EMRs) or untethered patient health records (PHRs) [4-6]. PHRs allow patients to *access, manage, and share* their complete longitudinal health data independently of any single institution [6]. Evidence suggests PHRs can reduce costs and improve outcomes by empowering self-care [4,7,8], yet utilization and satisfaction remain variable [9].

The NHS app, launched nationally in 2019, now exceeds 22  million registered users and represents most United Kingdom adults’ first exposure to a PHR ecosystem [10,11]. Previous research shows that activated patients can achieve higher levels of self-care and satisfaction by sharing all clinical notes with their clinical team, reducing their clinical workload, and improving the health information exchange [10,11]. A recent Canadian study surveyed national PHR users and highlighted time savings, reduced clinic visits, and high satisfaction [12]. They concluded that PHR users showed positive patient satisfaction, a measured decrease of appointment rates, and a decrease in their usage of the Canadian health system.

Comparable United Kingdom-wide data—including insights from individuals without PHR experience—are lacking. Understanding expectations and perceived utility among both groups is critical as PHR functions expand.

In the United Kingdom, digital access to personal health data has expanded rapidly. Approximately 73% of adults use the internet as their main health information source [13-15]. Despite the growing adoption of such tools, research on PHR user satisfaction in the United Kingdom remains limited and often focuses on small, nonrepresentative samples [16].

### Objectives

We sought to (1) identify determinants of PHR user satisfaction in a nationally representative sample of United Kingdom adults [17] and (2) compare perceptions between adults with real-world PHR experience and those expressing hypothetical expectations.

## Methods

### Design and Setting

We explicitly identify the study as a *descriptive, cross-sectional survey*. Data were collected online between 23‐25 March 2022. Informed electronic consent was obtained.

### Participants and Sampling

Because the survey assessed attitudes toward PHRs, it was essential to provide respondents with a clear understanding of what a PHR is. Accordingly, participants were shown a lay definition adapted from the Canadian study [12]. Approximately half of respondents had real experience with a PHR—most commonly the NHS app, which enables access to medical records, vaccination status, appointments, and prescriptions [18-20]. Other PHR systems integrated with the NHS App were also represented among users.

Participants were recruited via Prolific Academic [21], which maintains a United Kingdom participant panel. Using Prolific’s *nationally representative* option, we enforced quota stratification on age (six census bands), sex (male/female), and ethnicity (five ONS categories) to mirror 2021 United Kingdom Census proportions. Inclusion criteria were age ≥ 18 years and United Kingdom residence. A total of 1001 adults satisfied the quotas, resulting in margin-of-error±3 percentage-points at the 95% confidence level.

In addition to age, sex, and ethnicity, no further stratification variables (such as education level or household income) were available within the nationally representative Prolific panel profiles at the time of recruitment. Quotas were therefore restricted to Office for National Statistics census categories for age, sex, and ethnicity. The resulting sample distributions across these strata are reported in Multimedia Appendix 1.

### Survey Instrument

The questionnaire replicated the Canadian instrument [12] with United Kingdom contextual modifications (eg, “accident and emergency” replacing “emergency room”). Two parallel versions were administered: an experience-based version for respondents confirming prior PHR use and a hypothetical version with isomorphic wording framed as “If you had a PHR….” Sections covered (1) user satisfaction (5 items), (2) utility (12 items), (3) impact, and (4) demographics. The full instrument is now provided as a Survey Instrument Multimedia Appendix 2 .

### Statistical Analysis

Categorical variables (eg, gender, age group) were converted to dummy indicators. No missing data were observed. Between-group differences in satisfaction, utility, and impact outcomes were examined using independent two-sample t-tests with unequal variances (Multimedia Appendix 1). A multivariate analysis of variance (MANOVA) was conducted solely to assess demographic equivalence between respondents with and without prior PHR experience, using gender and age-band indicators as joint dependent variables. MANOVA was not used for outcome analysis.

All statistical tests used two-tailed p-values, with *P*<.05 considered significant. MANOVA assessed demographic equivalence across seven covariates (gender + six age bands). All analyses used STATA (version 17.0; StataCorp LLC), with *α*=.05.

### Ethical Considerations

The study is classified as a service evaluation per NHS REC guidance and is therefore exempt from formal NHS ethics review [22].

## Results

### Participant Characteristics

Data collection occurred between March 25, and April 5, 2022. A total of 1001 adults completed the survey (completion rate=99%). Table 1 compares sample demographics with 2021 Census benchmarks; deviations were <5  percentage points for all strata except adults aged ≥ 60 years (60‐69: −6 pp;≥70: −8 pp).

**Table 1.** Participant demographics by sample [experienced-based versus hypothetical].

Characteristics	Experience-based sample (n=468), n (%)	Hypothetical sample (n=533), n (%)	Nationally representative sample (2021), %
Gender			
Female	247 (53)	255 (48)	51
Male	217 (46)	263 (49)	49
Other	4 (1)	15 (3)	—^a^
Age			
18‐29	107 (23)	93 (17)	18
30‐39	93 (20)	100 (19)	17
40‐49	82 (18)	86 (16)	16
50‐59	86 (18)	114 (21)	18
60‐69	85 (18)	116 (22)	14
70+	15 (3)	24 (5)	17
Comfort with computers			
Completely comfortable	11 (2)	15 (3)	—
Comfortable	3 (1)	3 (1)	—
Neutral	9 (2)	22 (4)	—
Uncomfortable	112 (24)	185 (35)	—
Completely uncomfortable	333 (71)	308 (58)	—

a Not available.

A MANOVA assessing joint differences in sex and age distribution between respondents with and without prior PHR experience showed no statistically significant multivariate differences (Wilks’ Λ=0.99; F₆,₉₉₄=1.48; *P*=.18), indicating demographic balance between cohorts.

### Satisfaction, Impact and Utility

Among participants with PHR experience, 79% (370/468) agreed their PHR was easy to use and 68% (319/468) reported it saved time scheduling appointments. For those without experience, 99% (528/533) stated they would use a PHR to access personal health information and 72% (387/533) expected time savings. Ease of communication with care providers was reported by 60% (283/468) of experienced users versus 75% (398/533) of nonusers (*P*=.01).

PHR use reportedly avoided a clinic visit for 42% and an Accident and Emergency visit for 4% of experienced users. Hypothetical avoidance estimates were 32% and 9% among nonusers.

### Impact on Healthcare Use

Forty-two per cent (200/468) of experienced users indicated their PHR helped avoid at least one clinic visit, and 4% (19/468) avoided an emergency-room visit. Among nonusers, 32% (193/533) anticipated avoiding a clinic visit and 9% (47/533) an emergency visit.

### Preferred Features and Cost Savings

Experienced users most valued: viewing their own health information 81% (377/468); viewing lab results 50% (233/468); and scheduling appointments 47% (220/468). Nonusers prioritized: viewing health information 90% (479/533); lab results 76% (405/533); and appointment scheduling 58% (309/533). Cost savings most often related to time off work (40% (187/468) vs 57% (303/533), petrol/gasoline (30% (140/468) vs 52% (278/533), and parking (21% (98/468) vs 34% (181/533).

### Experience-Based vs Hypothetical Comparisons

Table 2 presents mean differences across 35 survey items. Nonusers significantly over-estimated future use for caregiving data access (eg, child records: Δ =+24  pp, *P*<.001). Despite these discrepancies, overall evaluation scores were similar (71 vs 74, *P*=.02). Specifically, three main differences emerged:

1. Nonusers over-estimated the extent to which they would use PHRs for family members’ data (eg, children: 36% vs 12%, *P*=.01).
2. They over-estimated available functionality, such as preclinic forms (55% vs 18%, *P*=.01).
3. Despite this, overall satisfaction scores were comparable (74 vs 71 out of 100, *P*=.01).

**Table 2.** Participant views on PHRs (experienced-based versus hypothetical).

Participant responses	Experience-based sample (n=468)	Hypothetical sample (n=533)
Requested improvement (scale 0 to 4), mean (SD)		—^a^
Less cumbersome login authentication	2.6 (1.0)	—
More data about my health	3.1 (0.8)	—
Access to lab and diagnostic imaging results	3.1 (0.8)	—
Access to data that would allow avoiding care visit	3.1 (0.8)	—
Question answered via secure message	2.9 (0.9)	—
General convenience and less waiting in clinic	3.2 (0.8)	—
Most preferred features, n (%)	n=468	n=533
Ease of communication with family’s care providers	68 (15)	165 (31)
Ease of scheduling appointments	220 (47)	309 (58)
Like to view own lab results	233 (50)	405 (76)
Like to view own health information	377 (81)	479 (90)
Like to view health information of carees	57 (12)	180 (34)
Like to fill out pre-appointment clinical questionnaire	83 (18)	291 (55)
Like to record blood pressure for care providers	31 (7)	151 (28)
Like to record glucose level for care providers	5 (1)	105 (20)
Other	17 (4)	16 (3)
Factors reported in self-reported avoidance of clinic visit, n (%)	n=468	n=533
Imaging results discussed by secure message	85 (18)	—
Question asked by message was answered	117 (25)	—
Other	20 (4)	—
Cost saving by avoiding clinic visit, n (%)	n=468	n= 533
Petrol (gasoline)	140 (30)	278 (52)
Time off work	187 (40)	3063 (57)
Getting childcare	32 (7)	70 (13)
Parking	98 (21)	181 (34)
Taxi	29 (6)	78 (15)
Other	28 (6)	65 (12)

a Not available.

Table 3 shows the sample mean for both groups across the different variables, along with the significance level from the aforementioned 2-sample *t*-test. There are three main findings associated with these results.

**Table 3.** Sample means by group [experienced-based versus hypothetical] with significance level of 2-sample t-test.

Variable	Hypothetical	Experience-based	Significance
Use PHR^a^ to access my health information	99%	96%	1%
Use PHR to access children’s health information	36%	12%	1%
Use PHR to access care health information	32%	9%	1%
Use PHR to access someone else’s health information	2%	4%	None
PHRs ease communication with family care providers	31%	15%	1%
PHRs ease scheduling appointments for self/family	58%	47%	1%
PHRs enable viewing own lab results	76%	50%	1%
PHRs enable viewing own health information	90%	81%	1%
PHRs enable viewing care health information	34%	12%	1%
PHRs enable filling out pre-appt clinical questionnaire	55%	18%	1%
PHRs enable recording BP for care providers	28%	7%	1%
PHRs enable recording glucose for care providers	20%	1%	1%
PHRs enable something else	3%	4%	None
PHRs are easy to use	76%	41%	1%
PHRs save time when scheduling appointments	87%	76%	10%
PHRs allow for more convenient communication with care providers	89%	61%	1%
PHR helpfulness rating 0‐100	74	71	5%
PHR benefit depends on volume of health data	1.00	1.01	None
PHR login frequency depends on volume of health data	0.47	0.72	1%
PHR benefit depends on promptness of data	0.96	0.93	None
PHR login frequency depends on promptness of data	0.58	0.71	5%
PHR has enabled self/family avoiding ER visit	9%	4%	1%
PHR has not enabled self/family avoiding clinic visit	64%	57%	5%
PHR allowing clinic visit avoidance saved petrol	52%	30%	1%
PHR allowing clinic visit avoidance saved work leave	57%	40%	1%
PHR allowing clinic visit avoidance saved childcare	13%	7%	1%
PHR allowing clinic visit avoidance saved parking	34%	21%	1%
PHR allowing clinic visit avoidance saved taxi	15%	6%	1%
PHR allowing clinic visit avoidance saved other	12%	6%	1%
PHR access from desktop/laptop	76%	51%	1%
PHR access from smartphone	64%	62%	None
PHR access from tablet	25%	12%	1%
PHR access no preference	6%	1%	1%

a PHR: patient health record.

## Discussion

### Principal Findings

This study examined factors influencing user satisfaction with PHRs among a nationally representative sample of adults in the United Kingdom. Two groups were compared—those with PHR experience and those without—to explore both lived and hypothetical perspectives on digital health record use. Demographically, the groups were comparable, indicating that observed differences in satisfaction and expectations primarily reflect PHR exposure rather than underlying population differences.

Overall, adults in the United Kingdom expressed strong support for access to their personal health information. Experienced users reported that PHRs were generally easy to use, time-saving, and useful for managing appointments, while nonusers anticipated similar benefits if they had access. These findings are consistent with evidence from other contexts demonstrating that PHR adoption enhances satisfaction, engagement, and communication with health care providers [4,6,12,13]. The results also mirror those from Canada [12], where PHR access was associated with improved efficiency and reduced appointment rates. However, users in the United Kingdom rated usability slightly lower than Canadian respondents, which may reflect differences in the maturity of the platforms, user training, and integration across healthcare systems [2,5,20].

### Comparison With Prior Work

Our results align with prior literature indicating that PHR usability, data accessibility, and perceived time savings are central determinants of satisfaction [3,4,6,17]. Participants with PHR experience valued the ability to view their own health and laboratory information, schedule appointments, and communicate with care providers—features frequently cited as key enablers of digital engagement [6,9,16]. These findings correspond with previous systematic reviews highlighting that patient portals improve convenience and empower users to manage their own health [4,5,7]. In contrast, our respondents rated United Kingdom systems slightly less favorably than those in comparable studies from Canada or the United States, suggesting ongoing challenges related to user interface design and interoperability within the NHS infrastructure [2,15,20].

Participants without prior experience tended to overestimate potential benefits and functionalities, a trend documented in earlier research on digital health optimism and hypothetical bias [10,15,16]. Woods et al [10] and Cunningham et al [16] found similar patterns of expectation inflation among new users of health portals, often due to limited understanding of actual system capabilities. Such insights underscore the importance of managing user expectations during onboarding and designing transparent communication strategies to clarify what PHRs can—and cannot—do.

In line with other studies, both experienced and hypothetical users viewed PHRs as tools that could reduce unnecessary clinical visits and associated costs such as travel, parking, and time off work [12,13,17]. This perception supports previous evidence that digital access to records can improve service efficiency and convenience for patients [4,7,9]. However, real-world data often indicate more modest effects, emphasizing the need for longitudinal studies assessing actual behavioral and economic outcomes [4,5].

Those with PHR experience expressed a desire for expanded functionality, greater interoperability, and simplified authentication—issues also raised by Turner et al [17] and Schneider et al [8], who highlighted the importance of usability and integration with existing clinical workflows. Improved personalisation, data richness, and accessibility were repeatedly mentioned, echoing prior calls for more inclusive and user-centered portal design [3,6,9,23].

From a policy perspective, the comparison between users and nonusers contributes new insights to the literature. While most previous studies focus solely on active users, our inclusion of a nationally representative sample allows for broader generalization. This approach complements earlier research that used local or condition-specific samples [8,16,17]. As digital transformation accelerates across the NHS, understanding perceptions among both users and nonusers is crucial for equitable and scalable implementation [15,18,20].

Finally, our findings align with recent studies demonstrating that both system and patient factors strongly predict portal use and satisfaction. Agrawal et al [24] reported that usability, system reliability, and patient demographics influence electronic medical record engagement. Ndabu et al [23] observed that access type and age group significantly affect portal breadth of use, highlighting digital literacy and inclusivity as enduring challenges. Moreover, Whittemore et al [25] showed that targeted interventions can increase patient portal use among adults with type 2 diabetes, particularly when support is localized and tailored to patient needs. Collectively, these studies reinforce our conclusion that user experience, accessibility, and system design remain the primary drivers of satisfaction and long-term adoption.

### Strengths and Limitations

Strengths include national representativeness and parallel assessment of users and nonusers. This is among the first nationally representative analyses of PHR satisfaction in the United Kingdom. It uniquely contrasts real and hypothetical users, offering insights into both actual experiences and potential barriers to uptake.

In terms of the limitations, first, the study’s cross-sectional design limits causal inference. Second, older age groups (60‐69 and ≥ 70 y) were under-represented in the sample, which may affect generalizability, particularly regarding digital literacy and accessibility. Third, hypothetical responses may reflect “hypothetical bias,” where participants without prior experience imagine more positive outcomes. Fourth, participation was voluntary, raising the possibility of self-selection bias toward more digitally engaged individuals. Finally, all measures were self-reported, and experience was not verified independently. Future research could employ longitudinal designs and link survey data with usage metrics to validate self-reports.

Second, education level and household income were not available as stratification or analytic variables within the nationally representative panel profiles used for recruitment. As socioeconomic status may influence digital access, health literacy, and engagement with patient-facing technologies, future studies should incorporate education and income measures where feasible to further refine equity-related insights.

### Conclusions

United Kingdom adults—both users and nonusers—demonstrate a strong interest in accessing their health data electronically. Experienced users report time and cost savings, while nonusers anticipate similar benefits. However, nonusers’ expectations often exceed current system capabilities, highlighting opportunities for continued system improvement and patient education. Ongoing evaluation of the NHS App and similar platforms will be crucial to achieving equitable, efficient, and user-centered digital health access in the United Kingdom.

## Supplementary material

10.2196/75935 Multimedia Appendix 1 Complete between‑group comparison tables (*t* tests).

10.2196/75935 Multimedia Appendix 2 Survey instrument.

## Abbreviations

EMR: electronic medical record
MANOVA: multivariate analysis of variance
NHS: National Health Service
PHR: patient health record
