# Supplementary material for: Factors Influencing User Satisfaction in Accessing Health Data: Cross-Sectional Survey of United Kingdom Adults
Source: JMIR Hum Factors. 2026 Apr 6;13:e75935. doi: 10.2196/75935 (PMC13052473; doi:10.2196/75935)
Supplement: Multimedia Appendix 1 [file humanfactors-v13-e75935-s001.docx]

Multimedia Appendix 2 reports independent two-sample t-tests with unequal variances comparing respondents with prior PHR experience (experienced cohort) and respondents without PHR experience (hypothetical cohort). These tests correspond to all satisfaction, utility, impact, access, and demographic variables reported in the Results section. Demographic equivalence across age and gender was assessed separately using MANOVA, with results reported in the main manuscript.

T Tests

*** A list of the variables that can be compared in real vs. hypothetical**

* phr_use_health_info_self phr_use_health_info_children phr_use_health_info_caree phr_use_health_info_other phr_like_comm_carer phr_like_schedule phr_like_lab_

results phr_like_health_info_self phr_like_health_info_carees phr_like_questionnaire phr_like_bp phr_like_glucose phr_like_other phr_eval_repeat_less phr_eval_

time_saving phr_eval_easy_comms phr_eval phr_benefit_data phr_login_data phr_benefit_promptness phr_login_promptness phr_avoid_er phr_avoid_clinic_no phr_savin

g_petrol phr_saving_leave phr_saving_childcare phr_saving_parking phr_saving_taxi phr_saving_other phr_access_desktop phr_access_mobile phr_access_tablet phr_a

ccess_indifferent gender_female gender_male gender_other age_18_29 age_30_39 age_40_49 age_50_59 age_60_69 age_70_plus computers_comfort_level carer_partner ca

rer_parents carer_children carer_other responsible_partner responsible_parents responsible_children responsible_other

**T-TEST OF REAL VS. HYPOTHETICAL VALUES OF phr_use_health_info_self**

**Two-sample t test with unequal variances**

------------------------------------------------------------------------------

Group | Obs Mean Std. Err. Std. Dev. [95% Conf. Interval]

---------+--------------------------------------------------------------------

0 | 533 .9906191 .0041794 .0964901 .9824089 .9988294

1 | 468 .9594017 .0091326 .1975689 .9414556 .9773478

---------+--------------------------------------------------------------------

combined | 1,001 .976024 .0048375 .1530509 .9665312 .9855167

---------+--------------------------------------------------------------------

diff | .0312174 .0100435 .0114962 .0509387

------------------------------------------------------------------------------

diff = mean(0) - mean(1) t = 3.1082

Ho: diff = 0 Satterthwaite's degrees of freedom = 657.769

Ha: diff < 0 Ha: diff != 0 Ha: diff > 0

Pr(T < t) = 0.9990 Pr(|T| > |t|) = 0.0020 Pr(T > t) = 0.0010

*********************************************

**T-TEST OF REAL VS. HYPOTHETICAL VALUES OF phr_use_health_info_children**

**Two-sample t test with unequal variances**

------------------------------------------------------------------------------

Group | Obs Mean Std. Err. Std. Dev. [95% Conf. Interval]

---------+--------------------------------------------------------------------

0 | 533 .3564728 .0207654 .4794071 .3156805 .3972651

1 | 468 .1217949 .015134 .3273987 .0920557 .1515341

---------+--------------------------------------------------------------------

combined | 1,001 .2467532 .0136333 .4313374 .2200001 .2735063

---------+--------------------------------------------------------------------

diff | .2346779 .0256952 .1842517 .2851042

------------------------------------------------------------------------------

diff = mean(0) - mean(1) t = 9.1332

Ho: diff = 0 Satterthwaite's degrees of freedom = 943.884

Ha: diff < 0 Ha: diff != 0 Ha: diff > 0

Pr(T < t) = 1.0000 Pr(|T| > |t|) = 0.0000 Pr(T > t) = 0.0000

*********************************************

**T-TEST OF REAL VS. HYPOTHETICAL VALUES OF phr_use_health_info_career**

**Two-sample t test with unequal variances**

------------------------------------------------------------------------------

Group | Obs Mean Std. Err. Std. Dev. [95% Conf. Interval]

---------+--------------------------------------------------------------------

0 | 533 .3208255 .0202381 .4672324 .2810692 .3605819

1 | 468 .0897436 .0132259 .2861199 .063754 .1157332

---------+--------------------------------------------------------------------

combined | 1,001 .2127872 .0129425 .409483 .1873896 .2381848

---------+--------------------------------------------------------------------

diff | .2310819 .0241765 .1836328 .2785311

------------------------------------------------------------------------------

diff = mean(0) - mean(1) t = 9.5581

Ho: diff = 0 Satterthwaite's degrees of freedom = 897.056

Ha: diff < 0 Ha: diff != 0 Ha: diff > 0

Pr(T < t) = 1.0000 Pr(|T| > |t|) = 0.0000 Pr(T > t) = 0.0000

*********************************************

**T-TEST OF REAL VS. HYPOTHETICAL VALUES OF phr_use_health_info_other**

**Two-sample t test with unequal variances**

------------------------------------------------------------------------------

Group | Obs Mean Std. Err. Std. Dev. [95% Conf. Interval]

---------+--------------------------------------------------------------------

0 | 533 .0243902 .0066879 .1544024 .0112523 .0375282

1 | 468 .0363248 .0086578 .1872972 .0193117 .0533379

---------+--------------------------------------------------------------------

combined | 1,001 .02997 .0053918 .1705899 .0193894 .0405506

---------+--------------------------------------------------------------------

diff | -.0119345 .0109401 -.0334054 .0095363

------------------------------------------------------------------------------

diff = mean(0) - mean(1) t = -1.0909

Ho: diff = 0 Satterthwaite's degrees of freedom = 907.09

Ha: diff < 0 Ha: diff != 0 Ha: diff > 0

Pr(T < t) = 0.1378 Pr(|T| > |t|) = 0.2756 Pr(T > t) = 0.8622

*********************************************

**T-TEST OF REAL VS. HYPOTHETICAL VALUES OF phr_like_comm_carer**

------------------------------------------------------------------------------

Group | Obs Mean Std. Err. Std. Dev. [95% Conf. Interval]

---------+--------------------------------------------------------------------

0 | 533 .3095685 .0200439 .46275 .2701935 .3489434

1 | 468 .1452991 .0163072 .3527793 .1132545 .1773438

---------+--------------------------------------------------------------------

combined | 1,001 .2327672 .0133636 .4228064 .2065433 .2589912

---------+--------------------------------------------------------------------

diff | .1642693 .0258396 .113562 .2149766

------------------------------------------------------------------------------

diff = mean(0) - mean(1) t = 6.3573

Ho: diff = 0 Satterthwaite's degrees of freedom = 980.153

Ha: diff < 0 Ha: diff != 0 Ha: diff > 0

Pr(T < t) = 1.0000 Pr(|T| > |t|) = 0.0000 Pr(T > t) = 0.0000

*********************************************

T-TEST OF REAL VS. HYPOTHETICAL VALUES OF phr_like_schedule

Two-sample t test with unequal variances

------------------------------------------------------------------------------

Group | Obs Mean Std. Err. Std. Dev. [95% Conf. Interval]

---------+--------------------------------------------------------------------

0 | 533 .5778612 .0214133 .4943644 .5357962 .6199262

1 | 468 .4722222 .0231015 .499762 .4268264 .517618

---------+--------------------------------------------------------------------

combined | 1,001 .5284715 .0157857 .4994382 .4974946 .5594485

---------+--------------------------------------------------------------------

diff | .1056389 .0314993 .043825 .1674529

------------------------------------------------------------------------------

diff = mean(0) - mean(1) t = 3.3537

Ho: diff = 0 Satterthwaite's degrees of freedom = 979.498

Ha: diff < 0 Ha: diff != 0 Ha: diff > 0

Pr(T < t) = 0.9996 Pr(|T| > |t|) = 0.0008 Pr(T > t) = 0.0004

*********************************************

T-TEST OF REAL VS. HYPOTHETICAL VALUES OF phr_like_lab_results

Two-sample t test with unequal variances

------------------------------------------------------------------------------

Group | Obs Mean Std. Err. Std. Dev. [95% Conf. Interval]

---------+--------------------------------------------------------------------

0 | 533 .7636023 .0184204 .4252683 .7274166 .7997879

1 | 468 .4978632 .023137 .5005305 .4523977 .5433288

---------+--------------------------------------------------------------------

combined | 1,001 .6393606 .0151848 .4804261 .6095629 .6691584

---------+--------------------------------------------------------------------

diff | .265739 .0295742 .2076984 .3237796

------------------------------------------------------------------------------

diff = mean(0) - mean(1) t = 8.9855

Ho: diff = 0 Satterthwaite's degrees of freedom = 921.608

Ha: diff < 0 Ha: diff != 0 Ha: diff > 0

Pr(T < t) = 1.0000 Pr(|T| > |t|) = 0.0000 Pr(T > t) = 0.0000

*********************************************

T-TEST OF REAL VS. HYPOTHETICAL VALUES OF phr_like_health_info_self

Two-sample t test with unequal variances

------------------------------------------------------------------------------

Group | Obs Mean Std. Err. Std. Dev. [95% Conf. Interval]

---------+--------------------------------------------------------------------

0 | 533 .8968105 .013189 .304492 .8709016 .9227194

1 | 468 .8055556 .0183142 .3961959 .7695672 .8415439

---------+--------------------------------------------------------------------

combined | 1,001 .8541459 .0111616 .3531364 .8322431 .8760486

---------+--------------------------------------------------------------------

diff | .091255 .022569 .046959 .1355509

------------------------------------------------------------------------------

diff = mean(0) - mean(1) t = 4.0434

Ho: diff = 0 Satterthwaite's degrees of freedom = 871.285

Ha: diff < 0 Ha: diff != 0 Ha: diff > 0

Pr(T < t) = 1.0000 Pr(|T| > |t|) = 0.0001 Pr(T > t) = 0.0000

*********************************************

T-TEST OF REAL VS. HYPOTHETICAL VALUES OF phr_like_health_info_carees

Two-sample t test with unequal variances

------------------------------------------------------------------------------

Group | Obs Mean Std. Err. Std. Dev. [95% Conf. Interval]

---------+--------------------------------------------------------------------

0 | 533 .3377111 .0205041 .4733738 .2974321 .37799

1 | 468 .1217949 .015134 .3273987 .0920557 .1515341

---------+--------------------------------------------------------------------

combined | 1,001 .2367632 .0134427 .4253083 .2103841 .2631424

---------+--------------------------------------------------------------------

diff | .2159162 .0254844 .1659038 .2659286

------------------------------------------------------------------------------

diff = mean(0) - mean(1) t = 8.4725

Ho: diff = 0 Satterthwaite's degrees of freedom = 948.767

Ha: diff < 0 Ha: diff != 0 Ha: diff > 0

Pr(T < t) = 1.0000 Pr(|T| > |t|) = 0.0000 Pr(T > t) = 0.0000

*********************************************

T-TEST OF REAL VS. HYPOTHETICAL VALUES OF phr_like_questionnaire

Two-sample t test with unequal variances

------------------------------------------------------------------------------

Group | Obs Mean Std. Err. Std. Dev. [95% Conf. Interval]

---------+--------------------------------------------------------------------

0 | 533 .5459662 .0215859 .4983503 .5035621 .5883704

1 | 468 .1773504 .0176752 .3823737 .1426176 .2120833

---------+--------------------------------------------------------------------

combined | 1,001 .3736264 .015298 .484008 .3436065 .4036463

---------+--------------------------------------------------------------------

diff | .3686158 .0278992 .3138668 .4233648

------------------------------------------------------------------------------

diff = mean(0) - mean(1) t = 13.2124

Ho: diff = 0 Satterthwaite's degrees of freedom = 981.771

Ha: diff < 0 Ha: diff != 0 Ha: diff > 0

Pr(T < t) = 1.0000 Pr(|T| > |t|) = 0.0000 Pr(T > t) = 0.0000

*********************************************

T-TEST OF REAL VS. HYPOTHETICAL VALUES OF phr_like_bp

Two-sample t test with unequal variances

------------------------------------------------------------------------------

Group | Obs Mean Std. Err. Std. Dev. [95% Conf. Interval]

---------+--------------------------------------------------------------------

0 | 533 .2833021 .0195361 .4510251 .2449248 .3216794

1 | 468 .0662393 .0115085 .2489661 .0436245 .0888541

---------+--------------------------------------------------------------------

combined | 1,001 .1818182 .0121967 .3858874 .1578841 .2057523

---------+--------------------------------------------------------------------

diff | .2170627 .0226738 .1725594 .2615661

------------------------------------------------------------------------------

diff = mean(0) - mean(1) t = 9.5733

Ho: diff = 0 Satterthwaite's degrees of freedom = 848.849

Ha: diff < 0 Ha: diff != 0 Ha: diff > 0

Pr(T < t) = 1.0000 Pr(|T| > |t|) = 0.0000 Pr(T > t) = 0.0000

*********************************************

T-TEST OF REAL VS. HYPOTHETICAL VALUES OF phr_like_glucose

Two-sample t test with unequal variances

------------------------------------------------------------------------------

Group | Obs Mean Std. Err. Std. Dev. [95% Conf. Interval]

---------+--------------------------------------------------------------------

0 | 533 .1969981 .0172438 .3981045 .1631238 .2308725

1 | 468 .0106838 .0047574 .1029187 .0013352 .0200324

---------+--------------------------------------------------------------------

combined | 1,001 .1098901 .0098901 .3129091 .0904824 .1292979

---------+--------------------------------------------------------------------

diff | .1863144 .0178881 .151185 .2214438

------------------------------------------------------------------------------

diff = mean(0) - mean(1) t = 10.4156

Ho: diff = 0 Satterthwaite's degrees of freedom = 612.03

Ha: diff < 0 Ha: diff != 0 Ha: diff > 0

Pr(T < t) = 1.0000 Pr(|T| > |t|) = 0.0000 Pr(T > t) = 0.0000

*********************************************

T-TEST OF REAL VS. HYPOTHETICAL VALUES OF phr_like_other

Two-sample t test with unequal variances

------------------------------------------------------------------------------

Group | Obs Mean Std. Err. Std. Dev. [95% Conf. Interval]

---------+--------------------------------------------------------------------

0 | 533 .0300188 .0073981 .1707992 .0154856 .0445519

1 | 468 .0363248 .0086578 .1872972 .0193117 .0533379

---------+--------------------------------------------------------------------

combined | 1,001 .032967 .0056463 .1786395 .0218872 .0440469

---------+--------------------------------------------------------------------

diff | -.006306 .0113882 -.0286548 .0160428

------------------------------------------------------------------------------

diff = mean(0) - mean(1) t = -0.5537

Ho: diff = 0 Satterthwaite's degrees of freedom = 952.285

Ha: diff < 0 Ha: diff != 0 Ha: diff > 0

Pr(T < t) = 0.2899 Pr(|T| > |t|) = 0.5799 Pr(T > t) = 0.7101

*********************************************

T-TEST OF REAL VS. HYPOTHETICAL VALUES OF phr_eval_repeat_less

Two-sample t test with unequal variances

------------------------------------------------------------------------------

Group | Obs Mean Std. Err. Std. Dev. [95% Conf. Interval]

---------+--------------------------------------------------------------------

0 | 533 .7617261 .0372163 .8592041 .6886172 .8348349

1 | 468 .4145299 .0420026 .908656 .3319923 .4970675

---------+--------------------------------------------------------------------

combined | 1,001 .5994006 .0284175 .8990882 .5436359 .6551653

---------+--------------------------------------------------------------------

diff | .3471962 .0561184 .2370681 .4573242

------------------------------------------------------------------------------

diff = mean(0) - mean(1) t = 6.1869

Ho: diff = 0 Satterthwaite's degrees of freedom = 965.643

Ha: diff < 0 Ha: diff != 0 Ha: diff > 0

Pr(T < t) = 1.0000 Pr(|T| > |t|) = 0.0000 Pr(T > t) = 0.0000

*********************************************

T-TEST OF REAL VS. HYPOTHETICAL VALUES OF phr_eval_time_saving

Two-sample t test with unequal variances

------------------------------------------------------------------------------

Group | Obs Mean Std. Err. Std. Dev. [95% Conf. Interval]

---------+--------------------------------------------------------------------

0 | 533 .8724203 .0387294 .8941377 .7963389 .9485016

1 | 468 .7606838 .0430578 .931482 .6760728 .8452947

---------+--------------------------------------------------------------------

combined | 1,001 .8201798 .0288583 .9130347 .7635501 .8768095

---------+--------------------------------------------------------------------

diff | .1117365 .0579132 -.001913 .225386

------------------------------------------------------------------------------

diff = mean(0) - mean(1) t = 1.9294

Ho: diff = 0 Satterthwaite's degrees of freedom = 970.626

Ha: diff < 0 Ha: diff != 0 Ha: diff > 0

Pr(T < t) = 0.9730 Pr(|T| > |t|) = 0.0540 Pr(T > t) = 0.0270

*********************************************

T-TEST OF REAL VS. HYPOTHETICAL VALUES OF phr_eval_easy_comms

Two-sample t test with unequal variances

------------------------------------------------------------------------------

Group | Obs Mean Std. Err. Std. Dev. [95% Conf. Interval]

---------+--------------------------------------------------------------------

0 | 533 .8893058 .0397692 .9181432 .8111819 .9674297

1 | 468 .6089744 .0395255 .8550672 .5313045 .6866442

---------+--------------------------------------------------------------------

combined | 1,001 .7582418 .0284374 .8997191 .7024379 .8140456

---------+--------------------------------------------------------------------

diff | .2803315 .0560701 .1703024 .3903606

------------------------------------------------------------------------------

diff = mean(0) - mean(1) t = 4.9997

Ho: diff = 0 Satterthwaite's degrees of freedom = 995.529

Ha: diff < 0 Ha: diff != 0 Ha: diff > 0

Pr(T < t) = 1.0000 Pr(|T| > |t|) = 0.0000 Pr(T > t) = 0.0000

*********************************************

T-TEST OF REAL VS. HYPOTHETICAL VALUES OF phr_eval

Two-sample t test with unequal variances

------------------------------------------------------------------------------

Group | Obs Mean Std. Err. Std. Dev. [95% Conf. Interval]

---------+--------------------------------------------------------------------

0 | 533 73.65103 .8775024 20.25872 71.92724 75.37483

1 | 468 70.89316 .8550288 18.4971 69.21298 72.57334

---------+--------------------------------------------------------------------

combined | 1,001 72.36164 .6161473 19.49403 71.15255 73.57073

---------+--------------------------------------------------------------------

diff | 2.75787 1.225188 .3536286 5.16211

------------------------------------------------------------------------------

diff = mean(0) - mean(1) t = 2.2510

Ho: diff = 0 Satterthwaite's degrees of freedom = 997.467

Ha: diff < 0 Ha: diff != 0 Ha: diff > 0

Pr(T < t) = 0.9877 Pr(|T| > |t|) = 0.0246 Pr(T > t) = 0.0123

*********************************************

T-TEST OF REAL VS. HYPOTHETICAL VALUES OF phr_benefit_data

Two-sample t test with unequal variances

------------------------------------------------------------------------------

Group | Obs Mean Std. Err. Std. Dev. [95% Conf. Interval]

---------+--------------------------------------------------------------------

0 | 533 1.001876 .0324724 .7496843 .9380863 1.065666

1 | 468 1.010684 .0337498 .7301207 .9443634 1.077004

---------+--------------------------------------------------------------------

combined | 1,001 1.005994 .0233969 .7402459 .9600813 1.051907

---------+--------------------------------------------------------------------

diff | -.0088076 .0468349 -.1007149 .0830997

------------------------------------------------------------------------------

diff = mean(0) - mean(1) t = -0.1881

Ho: diff = 0 Satterthwaite's degrees of freedom = 988.341

Ha: diff < 0 Ha: diff != 0 Ha: diff > 0

Pr(T < t) = 0.4254 Pr(|T| > |t|) = 0.8509 Pr(T > t) = 0.5746

*********************************************

T-TEST OF REAL VS. HYPOTHETICAL VALUES OF phr_login_data

Two-sample t test with unequal variances

------------------------------------------------------------------------------

Group | Obs Mean Std. Err. Std. Dev. [95% Conf. Interval]

---------+--------------------------------------------------------------------

0 | 533 .467167 .0410867 .9485599 .3864549 .547879

1 | 468 .715812 .0428451 .9268803 .631619 .8000049

---------+--------------------------------------------------------------------

combined | 1,001 .5834166 .0299064 .9461949 .5247302 .642103

---------+--------------------------------------------------------------------

diff | -.248645 .0593617 -.3651346 -.1321554

------------------------------------------------------------------------------

diff = mean(0) - mean(1) t = -4.1886

Ho: diff = 0 Satterthwaite's degrees of freedom = 987.656

Ha: diff < 0 Ha: diff != 0 Ha: diff > 0

Pr(T < t) = 0.0000 Pr(|T| > |t|) = 0.0000 Pr(T > t) = 1.0000

*********************************************

T-TEST OF REAL VS. HYPOTHETICAL VALUES OF phr_benefit_promptness

Two-sample t test with unequal variances

------------------------------------------------------------------------------

Group | Obs Mean Std. Err. Std. Dev. [95% Conf. Interval]

---------+--------------------------------------------------------------------

0 | 533 .956848 .0325272 .7509487 .8929505 1.020746

1 | 468 .9252137 .0331642 .7174522 .8600441 .9903833

---------+--------------------------------------------------------------------

combined | 1,001 .9420579 .02324 .7352818 .8964531 .9876628

---------+--------------------------------------------------------------------

diff | .0316344 .046453 -.0595232 .1227919

------------------------------------------------------------------------------

diff = mean(0) - mean(1) t = 0.6810

Ho: diff = 0 Satterthwaite's degrees of freedom = 991.895

Ha: diff < 0 Ha: diff != 0 Ha: diff > 0

Pr(T < t) = 0.7520 Pr(|T| > |t|) = 0.4960 Pr(T > t) = 0.2480

*********************************************

T-TEST OF REAL VS. HYPOTHETICAL VALUES OF phr_login_promptness

Two-sample t test with unequal variances

------------------------------------------------------------------------------

Group | Obs Mean Std. Err. Std. Dev. [95% Conf. Interval]

---------+--------------------------------------------------------------------

0 | 533 .575985 .0389495 .8992192 .4994713 .6524987

1 | 468 .7115385 .0399066 .8633108 .6331198 .7899571

---------+--------------------------------------------------------------------

combined | 1,001 .6393606 .0279647 .884765 .5844843 .694237

---------+--------------------------------------------------------------------

diff | -.1355535 .0557638 -.2449821 -.0261249

------------------------------------------------------------------------------

diff = mean(0) - mean(1) t = -2.4309

Ho: diff = 0 Satterthwaite's degrees of freedom = 991.057

Ha: diff < 0 Ha: diff != 0 Ha: diff > 0

Pr(T < t) = 0.0076 Pr(|T| > |t|) = 0.0152 Pr(T > t) = 0.9924

*********************************************

T-TEST OF REAL VS. HYPOTHETICAL VALUES OF phr_avoid_er

Two-sample t test with unequal variances

------------------------------------------------------------------------------

Group | Obs Mean Std. Err. Std. Dev. [95% Conf. Interval]

---------+--------------------------------------------------------------------

0 | 533 .0881801 .0122937 .283823 .0640299 .1123303

1 | 468 .0405983 .0091326 .1975689 .0226522 .0585444

---------+--------------------------------------------------------------------

combined | 1,001 .0659341 .0078477 .2482909 .0505342 .081334

---------+--------------------------------------------------------------------

diff | .0475818 .0153147 .0175273 .0776364

------------------------------------------------------------------------------

diff = mean(0) - mean(1) t = 3.1069

Ho: diff = 0 Satterthwaite's degrees of freedom = 951.191

Ha: diff < 0 Ha: diff != 0 Ha: diff > 0

Pr(T < t) = 0.9990 Pr(|T| > |t|) = 0.0019 Pr(T > t) = 0.0010

*********************************************

T-TEST OF REAL VS. HYPOTHETICAL VALUES OF phr_avoid_clinic_no

Two-sample t test with unequal variances

------------------------------------------------------------------------------

Group | Obs Mean Std. Err. Std. Dev. [95% Conf. Interval]

---------+--------------------------------------------------------------------

0 | 533 .6378987 .020837 .4810594 .5969658 .6788316

1 | 468 .5726496 .0228917 .4952233 .5276661 .6176331

---------+--------------------------------------------------------------------

combined | 1,001 .6073926 .0154424 .4885748 .5770894 .6376958

---------+--------------------------------------------------------------------

diff | .0652491 .030955 .004503 .1259952

------------------------------------------------------------------------------

diff = mean(0) - mean(1) t = 2.1079

Ho: diff = 0 Satterthwaite's degrees of freedom = 974.315

Ha: diff < 0 Ha: diff != 0 Ha: diff > 0

Pr(T < t) = 0.9824 Pr(|T| > |t|) = 0.0353 Pr(T > t) = 0.0176

*********************************************

T-TEST OF REAL VS. HYPOTHETICAL VALUES OF phr_saving_petrol

Two-sample t test with unequal variances

------------------------------------------------------------------------------

Group | Obs Mean Std. Err. Std. Dev. [95% Conf. Interval]

---------+--------------------------------------------------------------------

0 | 533 .521576 .0216576 .5000035 .4790312 .5641208

1 | 468 .2991453 .0211883 .4583736 .257509 .3407816

---------+--------------------------------------------------------------------

combined | 1,001 .4175824 .0155951 .4934071 .3869795 .4481853

---------+--------------------------------------------------------------------

diff | .2224307 .0302984 .1629747 .2818867

------------------------------------------------------------------------------

diff = mean(0) - mean(1) t = 7.3413

Ho: diff = 0 Satterthwaite's degrees of freedom = 997.134

Ha: diff < 0 Ha: diff != 0 Ha: diff > 0

Pr(T < t) = 1.0000 Pr(|T| > |t|) = 0.0000 Pr(T > t) = 0.0000

*********************************************

T-TEST OF REAL VS. HYPOTHETICAL VALUES OF phr_saving_leave

Two-sample t test with unequal variances

------------------------------------------------------------------------------

Group | Obs Mean Std. Err. Std. Dev. [95% Conf. Interval]

---------+--------------------------------------------------------------------

0 | 533 .5741088 .0214383 .4949419 .5319947 .616223

1 | 468 .3952991 .0226243 .489438 .3508411 .4397571

---------+--------------------------------------------------------------------

combined | 1,001 .4905095 .0158085 .5001598 .4594878 .5215312

---------+--------------------------------------------------------------------

diff | .1788097 .0311682 .1176459 .2399735

------------------------------------------------------------------------------

diff = mean(0) - mean(1) t = 5.7369

Ho: diff = 0 Satterthwaite's degrees of freedom = 985.025

Ha: diff < 0 Ha: diff != 0 Ha: diff > 0

Pr(T < t) = 1.0000 Pr(|T| > |t|) = 0.0000 Pr(T > t) = 0.0000

*********************************************

T-TEST OF REAL VS. HYPOTHETICAL VALUES OF phr_saving_childcare

Two-sample t test with unequal variances

------------------------------------------------------------------------------

Group | Obs Mean Std. Err. Std. Dev. [95% Conf. Interval]

---------+--------------------------------------------------------------------

0 | 533 .1313321 .0146439 .3380805 .1025651 .160099

1 | 468 .0683761 .0116792 .2526602 .0454257 .0913264

---------+--------------------------------------------------------------------

combined | 1,001 .1018981 .0095663 .3026655 .0831257 .1206705

---------+--------------------------------------------------------------------

diff | .062956 .0187309 .0261984 .0997136

------------------------------------------------------------------------------

diff = mean(0) - mean(1) t = 3.3611

Ho: diff = 0 Satterthwaite's degrees of freedom = 974.759

Ha: diff < 0 Ha: diff != 0 Ha: diff > 0

Pr(T < t) = 0.9996 Pr(|T| > |t|) = 0.0008 Pr(T > t) = 0.0004

*********************************************

T-TEST OF REAL VS. HYPOTHETICAL VALUES OF phr_saving_parking

Two-sample t test with unequal variances

------------------------------------------------------------------------------

Group | Obs Mean Std. Err. Std. Dev. [95% Conf. Interval]

---------+--------------------------------------------------------------------

0 | 533 .3414634 .0205592 .474646 .3010762 .3818506

1 | 468 .2136752 .0189679 .4103388 .1764022 .2509482

---------+--------------------------------------------------------------------

combined | 1,001 .2817183 .0142251 .4500616 .2538038 .3096327

---------+--------------------------------------------------------------------

diff | .1277882 .0279725 .0728965 .1826799

------------------------------------------------------------------------------

diff = mean(0) - mean(1) t = 4.5683

Ho: diff = 0 Satterthwaite's degrees of freedom = 998.765

Ha: diff < 0 Ha: diff != 0 Ha: diff > 0

Pr(T < t) = 1.0000 Pr(|T| > |t|) = 0.0000 Pr(T > t) = 0.0000

*********************************************

T-TEST OF REAL VS. HYPOTHETICAL VALUES OF phr_saving_taxi

Two-sample t test with unequal variances

------------------------------------------------------------------------------

Group | Obs Mean Std. Err. Std. Dev. [95% Conf. Interval]

---------+--------------------------------------------------------------------

0 | 533 .1463415 .0153239 .3537802 .1162386 .1764443

1 | 468 .0619658 .0111565 .2413514 .0400427 .0838889

---------+--------------------------------------------------------------------

combined | 1,001 .1068931 .0097707 .3091317 .0877196 .1260666

---------+--------------------------------------------------------------------

diff | .0843757 .0189549 .0471769 .1215744

------------------------------------------------------------------------------

diff = mean(0) - mean(1) t = 4.4514

Ho: diff = 0 Satterthwaite's degrees of freedom = 943.473

Ha: diff < 0 Ha: diff != 0 Ha: diff > 0

Pr(T < t) = 1.0000 Pr(|T| > |t|) = 0.0000 Pr(T > t) = 0.0000

*********************************************

T-TEST OF REAL VS. HYPOTHETICAL VALUES OF phr_saving_other

Two-sample t test with unequal variances

------------------------------------------------------------------------------

Group | Obs Mean Std. Err. Std. Dev. [95% Conf. Interval]

---------+--------------------------------------------------------------------

0 | 533 .1219512 .0141872 .3275369 .0940814 .149821

1 | 468 .0598291 .0109749 .2374237 .0382627 .0813954

---------+--------------------------------------------------------------------

combined | 1,001 .0929071 .0091802 .2904473 .0748925 .1109217

---------+--------------------------------------------------------------------

diff | .0621222 .0179367 .0269227 .0973216

------------------------------------------------------------------------------

diff = mean(0) - mean(1) t = 3.4634

Ho: diff = 0 Satterthwaite's degrees of freedom = 965.399

Ha: diff < 0 Ha: diff != 0 Ha: diff > 0

Pr(T < t) = 0.9997 Pr(|T| > |t|) = 0.0006 Pr(T > t) = 0.0003

*********************************************

T-TEST OF REAL VS. HYPOTHETICAL VALUES OF phr_access_desktop

Two-sample t test with unequal variances

------------------------------------------------------------------------------

Group | Obs Mean Std. Err. Std. Dev. [95% Conf. Interval]

---------+--------------------------------------------------------------------

0 | 533 .7560976 .0186183 .429838 .7195231 .7926721

1 | 468 .5064103 .0231353 .5004939 .460948 .5518725

---------+--------------------------------------------------------------------

combined | 1,001 .6393606 .0151848 .4804261 .6095629 .6691584

---------+--------------------------------------------------------------------

diff | .2496873 .0296966 .191407 .3079677

------------------------------------------------------------------------------

diff = mean(0) - mean(1) t = 8.4079

Ho: diff = 0 Satterthwaite's degrees of freedom = 926.604

Ha: diff < 0 Ha: diff != 0 Ha: diff > 0

Pr(T < t) = 1.0000 Pr(|T| > |t|) = 0.0000 Pr(T > t) = 0.0000

*********************************************

T-TEST OF REAL VS. HYPOTHETICAL VALUES OF phr_access_mobile

Two-sample t test with unequal variances

------------------------------------------------------------------------------

Group | Obs Mean Std. Err. Std. Dev. [95% Conf. Interval]

---------+--------------------------------------------------------------------

0 | 533 .6397749 .0208135 .4805166 .5988882 .6806616

1 | 468 .6239316 .0224152 .4849159 .5798844 .6679789

---------+--------------------------------------------------------------------

combined | 1,001 .6323676 .0152473 .4824016 .6024473 .6622879

---------+--------------------------------------------------------------------

diff | .0158432 .0305883 -.0441829 .0758693

------------------------------------------------------------------------------

diff = mean(0) - mean(1) t = 0.5180

Ho: diff = 0 Satterthwaite's degrees of freedom = 979.966

Ha: diff < 0 Ha: diff != 0 Ha: diff > 0

Pr(T < t) = 0.6977 Pr(|T| > |t|) = 0.6046 Pr(T > t) = 0.3023

*********************************************

T-TEST OF REAL VS. HYPOTHETICAL VALUES OF phr_access_tablet

Two-sample t test with unequal variances

------------------------------------------------------------------------------

Group | Obs Mean Std. Err. Std. Dev. [95% Conf. Interval]

---------+--------------------------------------------------------------------

0 | 533 .2457786 .0186666 .4309524 .2091093 .2824479

1 | 468 .1175214 .0149023 .3223851 .0882376 .1468052

---------+--------------------------------------------------------------------

combined | 1,001 .1858142 .0122999 .3891511 .1616776 .2099507

---------+--------------------------------------------------------------------

diff | .1282572 .0238856 .0813842 .1751303

------------------------------------------------------------------------------

diff = mean(0) - mean(1) t = 5.3697

Ho: diff = 0 Satterthwaite's degrees of freedom = 975.04

Ha: diff < 0 Ha: diff != 0 Ha: diff > 0

Pr(T < t) = 1.0000 Pr(|T| > |t|) = 0.0000 Pr(T > t) = 0.0000

*********************************************

T-TEST OF REAL VS. HYPOTHETICAL VALUES OF phr_access_indifferent

Two-sample t test with unequal variances

------------------------------------------------------------------------------

Group | Obs Mean Std. Err. Std. Dev. [95% Conf. Interval]

---------+--------------------------------------------------------------------

0 | 533 .0637899 .0105951 .2446078 .0429764 .0846033

1 | 468 .0106838 .0047574 .1029187 .0013352 .0200324

---------+--------------------------------------------------------------------

combined | 1,001 .038961 .0061191 .1935989 .0269533 .0509687

---------+--------------------------------------------------------------------

diff | .0531061 .0116142 .0303051 .0759071

------------------------------------------------------------------------------

diff = mean(0) - mean(1) t = 4.5725

Ho: diff = 0 Satterthwaite's degrees of freedom = 734.15

Ha: diff < 0 Ha: diff != 0 Ha: diff > 0

Pr(T < t) = 1.0000 Pr(|T| > |t|) = 0.0000 Pr(T > t) = 0.0000

*********************************************

T-TEST OF REAL VS. HYPOTHETICAL VALUES OF gender_female

Two-sample t test with unequal variances

------------------------------------------------------------------------------

Group | Obs Mean Std. Err. Std. Dev. [95% Conf. Interval]

---------+--------------------------------------------------------------------

0 | 533 .478424 .0216576 .5000035 .4358792 .5209688

1 | 468 .5277778 .0231015 .499762 .482382 .5731736

---------+--------------------------------------------------------------------

combined | 1,001 .5014985 .0158113 .5002477 .4704713 .5325257

---------+--------------------------------------------------------------------

diff | -.0493538 .0316659 -.1114943 .0127868

------------------------------------------------------------------------------

diff = mean(0) - mean(1) t = -1.5586

Ho: diff = 0 Satterthwaite's degrees of freedom = 982.449

Ha: diff < 0 Ha: diff != 0 Ha: diff > 0

Pr(T < t) = 0.0597 Pr(|T| > |t|) = 0.1194 Pr(T > t) = 0.9403

*********************************************

T-TEST OF REAL VS. HYPOTHETICAL VALUES OF gender_male

Two-sample t test with unequal variances

------------------------------------------------------------------------------

Group | Obs Mean Std. Err. Std. Dev. [95% Conf. Interval]

---------+--------------------------------------------------------------------

0 | 533 .4934334 .0216759 .5004265 .4508526 .5360142

1 | 468 .4636752 .0230761 .4992124 .4183294 .5090211

---------+--------------------------------------------------------------------

combined | 1,001 .4795205 .0157981 .4998301 .4485192 .5105217

---------+--------------------------------------------------------------------

diff | .0297582 .0316599 -.0323706 .091887

------------------------------------------------------------------------------

diff = mean(0) - mean(1) t = 0.9399

Ho: diff = 0 Satterthwaite's degrees of freedom = 982.933

Ha: diff < 0 Ha: diff != 0 Ha: diff > 0

Pr(T < t) = 0.8263 Pr(|T| > |t|) = 0.3475 Pr(T > t) = 0.1737

*********************************************

T-TEST OF REAL VS. HYPOTHETICAL VALUES OF gender_other

Two-sample t test with unequal variances

------------------------------------------------------------------------------

Group | Obs Mean Std. Err. Std. Dev. [95% Conf. Interval]

---------+--------------------------------------------------------------------

0 | 533 .0281426 .0071701 .1655355 .0140573 .0422278

1 | 468 .008547 .0042598 .0921526 .0001763 .0169177

---------+--------------------------------------------------------------------

combined | 1,001 .018981 .0043152 .136526 .0105132 .0274489

---------+--------------------------------------------------------------------

diff | .0195956 .00834 .0032262 .035965

------------------------------------------------------------------------------

diff = mean(0) - mean(1) t = 2.3496

Ho: diff = 0 Satterthwaite's degrees of freedom = 852.791

Ha: diff < 0 Ha: diff != 0 Ha: diff > 0

Pr(T < t) = 0.9905 Pr(|T| > |t|) = 0.0190 Pr(T > t) = 0.0095

*********************************************

T-TEST OF REAL VS. HYPOTHETICAL VALUES OF age_18_29

Two-sample t test with unequal variances

------------------------------------------------------------------------------

Group | Obs Mean Std. Err. Std. Dev. [95% Conf. Interval]

---------+--------------------------------------------------------------------

0 | 533 .1744841 .0164545 .3798817 .1421603 .2068078

1 | 468 .2286325 .0194331 .4204014 .1904454 .2668195

---------+--------------------------------------------------------------------

combined | 1,001 .1998002 .0126444 .4000499 .1749877 .2246127

---------+--------------------------------------------------------------------

diff | -.0541484 .0254636 -.1041199 -.0041769

------------------------------------------------------------------------------

diff = mean(0) - mean(1) t = -2.1265

Ho: diff = 0 Satterthwaite's degrees of freedom = 948.637

Ha: diff < 0 Ha: diff != 0 Ha: diff > 0

Pr(T < t) = 0.0169 Pr(|T| > |t|) = 0.0337 Pr(T > t) = 0.9831

*********************************************

T-TEST OF REAL VS. HYPOTHETICAL VALUES OF age_30_39

Two-sample t test with unequal variances

------------------------------------------------------------------------------

Group | Obs Mean Std. Err. Std. Dev. [95% Conf. Interval]

---------+--------------------------------------------------------------------

0 | 533 .1876173 .0169263 .390773 .1543668 .2208678

1 | 468 .1987179 .0184651 .3994622 .1624329 .235003

---------+--------------------------------------------------------------------

combined | 1,001 .1928072 .0124753 .3947002 .1683265 .2172879

---------+--------------------------------------------------------------------

diff | -.0111007 .0250491 -.0602571 .0380557

------------------------------------------------------------------------------

diff = mean(0) - mean(1) t = -0.4432

Ho: diff = 0 Satterthwaite's degrees of freedom = 976.386

Ha: diff < 0 Ha: diff != 0 Ha: diff > 0

Pr(T < t) = 0.3289 Pr(|T| > |t|) = 0.6578 Pr(T > t) = 0.6711

*********************************************

T-TEST OF REAL VS. HYPOTHETICAL VALUES OF age_40_49

Two-sample t test with unequal variances

------------------------------------------------------------------------------

Group | Obs Mean Std. Err. Std. Dev. [95% Conf. Interval]

---------+--------------------------------------------------------------------

0 | 533 .1613508 .0159485 .3681998 .1300211 .1926806

1 | 468 .1752137 .0175912 .3805566 .1406459 .2097814

---------+--------------------------------------------------------------------

combined | 1,001 .1678322 .011818 .373904 .1446413 .191023

---------+--------------------------------------------------------------------

diff | -.0138628 .0237446 -.0604594 .0327337

------------------------------------------------------------------------------

diff = mean(0) - mean(1) t = -0.5838

Ho: diff = 0 Satterthwaite's degrees of freedom = 973.103

Ha: diff < 0 Ha: diff != 0 Ha: diff > 0

Pr(T < t) = 0.2797 Pr(|T| > |t|) = 0.5595 Pr(T > t) = 0.7203

*********************************************

T-TEST OF REAL VS. HYPOTHETICAL VALUES OF age_50_59

Two-sample t test with unequal variances

------------------------------------------------------------------------------

Group | Obs Mean Std. Err. Std. Dev. [95% Conf. Interval]

---------+--------------------------------------------------------------------

0 | 533 .2138837 .0177777 .4104309 .1789605 .2488068

1 | 468 .1837607 .0179216 .3877033 .1485437 .2189776

---------+--------------------------------------------------------------------

combined | 1,001 .1998002 .0126444 .4000499 .1749877 .2246127

---------+--------------------------------------------------------------------

diff | .030123 .0252434 -.0194136 .0796596

------------------------------------------------------------------------------

diff = mean(0) - mean(1) t = 1.1933

Ho: diff = 0 Satterthwaite's degrees of freedom = 993.664

Ha: diff < 0 Ha: diff != 0 Ha: diff > 0

Pr(T < t) = 0.8835 Pr(|T| > |t|) = 0.2330 Pr(T > t) = 0.1165

*********************************************

T-TEST OF REAL VS. HYPOTHETICAL VALUES OF age_60_69

Two-sample t test with unequal variances

------------------------------------------------------------------------------

Group | Obs Mean Std. Err. Std. Dev. [95% Conf. Interval]

---------+--------------------------------------------------------------------

0 | 533 .217636 .0178902 .4130262 .182492 .25278

1 | 468 .1816239 .0178404 .3859468 .1465665 .2166813

---------+--------------------------------------------------------------------

combined | 1,001 .2007992 .012668 .4007984 .1759402 .2256582

---------+--------------------------------------------------------------------

diff | .0360121 .0252653 -.0135674 .0855915

------------------------------------------------------------------------------

diff = mean(0) - mean(1) t = 1.4254

Ho: diff = 0 Satterthwaite's degrees of freedom = 995.124

Ha: diff < 0 Ha: diff != 0 Ha: diff > 0

Pr(T < t) = 0.9228 Pr(|T| > |t|) = 0.1544 Pr(T > t) = 0.0772

*********************************************

T-TEST OF REAL VS. HYPOTHETICAL VALUES OF age_70_plus

Two-sample t test with unequal variances

------------------------------------------------------------------------------

Group | Obs Mean Std. Err. Std. Dev. [95% Conf. Interval]

---------+--------------------------------------------------------------------

0 | 533 .0450281 .0089905 .2075607 .027367 .0626893

1 | 468 .0320513 .0081506 .1763248 .0160349 .0480677

---------+--------------------------------------------------------------------

combined | 1,001 .038961 .0061191 .1935989 .0269533 .0509687

---------+--------------------------------------------------------------------

diff | .0129769 .0121351 -.0108364 .0367901

------------------------------------------------------------------------------

diff = mean(0) - mean(1) t = 1.0694

Ho: diff = 0 Satterthwaite's degrees of freedom = 997.929

Ha: diff < 0 Ha: diff != 0 Ha: diff > 0

Pr(T < t) = 0.8574 Pr(|T| > |t|) = 0.2852 Pr(T > t) = 0.1426

*********************************************

T-TEST OF REAL VS. HYPOTHETICAL VALUES OF computers_comfort_level

Two-sample t test with unequal variances

------------------------------------------------------------------------------

Group | Obs Mean Std. Err. Std. Dev. [95% Conf. Interval]

---------+--------------------------------------------------------------------

0 | 533 3.440901 .0362878 .8377696 3.369616 3.512186

1 | 468 3.608974 .0357573 .7735487 3.538709 3.679239

---------+--------------------------------------------------------------------

combined | 1,001 3.519481 .0256751 .8123239 3.469097 3.569864

---------+--------------------------------------------------------------------

diff | -.1680738 .050945 -.2680455 -.068102

------------------------------------------------------------------------------

diff = mean(0) - mean(1) t = -3.2991

Ho: diff = 0 Satterthwaite's degrees of freedom = 996.464

Ha: diff < 0 Ha: diff != 0 Ha: diff > 0

Pr(T < t) = 0.0005 Pr(|T| > |t|) = 0.0010 Pr(T > t) = 0.9995

*********************************************

T-TEST OF REAL VS. HYPOTHETICAL VALUES OF carer_partner

Two-sample t test with unequal variances

------------------------------------------------------------------------------

Group | Obs Mean Std. Err. Std. Dev. [95% Conf. Interval]

---------+--------------------------------------------------------------------

0 | 533 .5684803 .0214735 .4957535 .5262971 .6106635

1 | 468 .1495726 .0165039 .3570337 .1171416 .1820037

---------+--------------------------------------------------------------------

combined | 1,001 .3726274 .0152897 .4837458 .3426237 .402631

---------+--------------------------------------------------------------------

diff | .4189077 .027083 .3657592 .4720561

------------------------------------------------------------------------------

diff = mean(0) - mean(1) t = 15.4676

Ho: diff = 0 Satterthwaite's degrees of freedom = 963.25

Ha: diff < 0 Ha: diff != 0 Ha: diff > 0

Pr(T < t) = 1.0000 Pr(|T| > |t|) = 0.0000 Pr(T > t) = 0.0000

*********************************************

T-TEST OF REAL VS. HYPOTHETICAL VALUES OF carer_parents

Two-sample t test with unequal variances

------------------------------------------------------------------------------

Group | Obs Mean Std. Err. Std. Dev. [95% Conf. Interval]

---------+--------------------------------------------------------------------

0 | 533 .2213884 .0180004 .4155714 .1860278 .2567489

1 | 468 .0641026 .0113343 .2451977 .0418301 .086375

---------+--------------------------------------------------------------------

combined | 1,001 .1478521 .0112246 .3551308 .1258257 .1698786

---------+--------------------------------------------------------------------

diff | .1572858 .0212716 .1155369 .1990347

------------------------------------------------------------------------------

diff = mean(0) - mean(1) t = 7.3942

Ho: diff = 0 Satterthwaite's degrees of freedom = 879.913

Ha: diff < 0 Ha: diff != 0 Ha: diff > 0

Pr(T < t) = 1.0000 Pr(|T| > |t|) = 0.0000 Pr(T > t) = 0.0000

*********************************************

T-TEST OF REAL VS. HYPOTHETICAL VALUES OF carer_children

Two-sample t test with unequal variances

------------------------------------------------------------------------------

Group | Obs Mean Std. Err. Std. Dev. [95% Conf. Interval]

---------+--------------------------------------------------------------------

0 | 533 .2682927 .0192096 .443487 .2305568 .3060286

1 | 468 .1260684 .0153597 .3322816 .0958856 .1562511

---------+--------------------------------------------------------------------

combined | 1,001 .2017982 .0126916 .401543 .1768931 .2267033

---------+--------------------------------------------------------------------

diff | .1422243 .0245953 .0939585 .1904901

------------------------------------------------------------------------------

diff = mean(0) - mean(1) t = 5.7826

Ho: diff = 0 Satterthwaite's degrees of freedom = 975.483

Ha: diff < 0 Ha: diff != 0 Ha: diff > 0

Pr(T < t) = 1.0000 Pr(|T| > |t|) = 0.0000 Pr(T > t) = 0.0000

*********************************************

T-TEST OF REAL VS. HYPOTHETICAL VALUES OF carer_other

Two-sample t test with unequal variances

------------------------------------------------------------------------------

Group | Obs Mean Std. Err. Std. Dev. [95% Conf. Interval]

---------+--------------------------------------------------------------------

0 | 533 .0506567 .0095077 .2195016 .0319795 .0693338

1 | 468 .0576923 .0107894 .2334102 .0364905 .0788941

---------+--------------------------------------------------------------------

combined | 1,001 .0539461 .0071439 .2260241 .0399272 .0679649

---------+--------------------------------------------------------------------

diff | -.0070356 .0143808 -.0352569 .0211856

------------------------------------------------------------------------------

diff = mean(0) - mean(1) t = -0.4892

Ho: diff = 0 Satterthwaite's degrees of freedom = 963.744

Ha: diff < 0 Ha: diff != 0 Ha: diff > 0

Pr(T < t) = 0.3124 Pr(|T| > |t|) = 0.6248 Pr(T > t) = 0.6876

*********************************************

T-TEST OF REAL VS. HYPOTHETICAL VALUES OF responsible_partner

Two-sample t test with unequal variances

------------------------------------------------------------------------------

Group | Obs Mean Std. Err. Std. Dev. [95% Conf. Interval]

---------+--------------------------------------------------------------------

0 | 533 .1106942 .0136029 .3140478 .0839722 .1374162

1 | 468 .0961538 .0136418 .2951176 .0693469 .1229608

---------+--------------------------------------------------------------------

combined | 1,001 .1038961 .0096489 .3052782 .0849616 .1228306

---------+--------------------------------------------------------------------

diff | .0145403 .019265 -.0232643 .052345

------------------------------------------------------------------------------

diff = mean(0) - mean(1) t = 0.7548

Ho: diff = 0 Satterthwaite's degrees of freedom = 994.393

Ha: diff < 0 Ha: diff != 0 Ha: diff > 0

Pr(T < t) = 0.7747 Pr(|T| > |t|) = 0.4506 Pr(T > t) = 0.2253

*********************************************

T-TEST OF REAL VS. HYPOTHETICAL VALUES OF responsible_parents

Two-sample t test with unequal variances

------------------------------------------------------------------------------

Group | Obs Mean Std. Err. Std. Dev. [95% Conf. Interval]

---------+--------------------------------------------------------------------

0 | 533 .0863039 .0121748 .2810761 .0623874 .1102204

1 | 468 .0854701 .0129374 .2798791 .0600473 .1108928

---------+--------------------------------------------------------------------

combined | 1,001 .0859141 .0088619 .2803772 .0685241 .1033041

---------+--------------------------------------------------------------------

diff | .0008339 .0177652 -.0340282 .0356959

------------------------------------------------------------------------------

diff = mean(0) - mean(1) t = 0.0469

Ho: diff = 0 Satterthwaite's degrees of freedom = 983.384

Ha: diff < 0 Ha: diff != 0 Ha: diff > 0

Pr(T < t) = 0.5187 Pr(|T| > |t|) = 0.9626 Pr(T > t) = 0.4813

*********************************************

T-TEST OF REAL VS. HYPOTHETICAL VALUES OF responsible_children

Two-sample t test with unequal variances

------------------------------------------------------------------------------

Group | Obs Mean Std. Err. Std. Dev. [95% Conf. Interval]

---------+--------------------------------------------------------------------

0 | 533 .2307692 .0182668 .4217208 .1948854 .2666531

1 | 468 .2200855 .0191717 .4147474 .182412 .257759

---------+--------------------------------------------------------------------

combined | 1,001 .2257742 .0132212 .4183002 .1998297 .2517187

---------+--------------------------------------------------------------------

diff | .0106838 .0264807 -.0412813 .0626488

------------------------------------------------------------------------------

diff = mean(0) - mean(1) t = 0.4035

Ho: diff = 0 Satterthwaite's degrees of freedom = 986.268

Ha: diff < 0 Ha: diff != 0 Ha: diff > 0

Pr(T < t) = 0.6566 Pr(|T| > |t|) = 0.6867 Pr(T > t) = 0.3434

*********************************************

T-TEST OF REAL VS. HYPOTHETICAL VALUES OF responsible_other

Two-sample t test with unequal variances

------------------------------------------------------------------------------

Group | Obs Mean Std. Err. Std. Dev. [95% Conf. Interval]

---------+--------------------------------------------------------------------

0 | 533 .0356473 .0080385 .1855833 .0198562 .0514384

1 | 468 .0448718 .0095799 .207244 .0260468 .0636968

---------+--------------------------------------------------------------------

combined | 1,001 .03996 .0061938 .1959633 .0278057 .0521144

---------+--------------------------------------------------------------------

diff | -.0092245 .0125056 -.0337666 .0153175

------------------------------------------------------------------------------

diff = mean(0) - mean(1) t = -0.7376

Ho: diff = 0 Satterthwaite's degrees of freedom = 944.928

Ha: diff < 0 Ha: diff != 0 Ha: diff > 0

Pr(T < t) = 0.2305 Pr(|T| > |t|) = 0.4609 Pr(T > t) = 0.7695

*********************************************

Note: Group coding follows phr_experience = 1 (experienced) and phr_experience = 0 (hypothetical). All tests use two-tailed p-values with significance set at p < 0.05.
