# Supplementary material for: Factors Influencing User Satisfaction in Accessing Health Data: Cross-Sectional Survey of United Kingdom Adults
Source: JMIR Hum Factors. 2026 Apr 6;13:e75935. doi: 10.2196/75935 (PMC13052473; doi:10.2196/75935)
Supplement: Multimedia Appendix 2 [file humanfactors-v13-e75935-s002.docx]

Multimedia Appendix 1 presents the full survey instrument administered to respondents. Two parallel versions were used: an experience-based version for respondents with prior PHR use and a hypothetical version with isomorphic wording for respondents without prior experience.

**Survey Questionnaire on Patients Portals or PHRs**

This survey is about patient health records. Participating takes approximately 8 minutes. Are you at least 18 years of age?

a)Yes

b)No

* Please enter your Prolific ID

|  |
| --- |

Screening question

* A patient health record (PHR) or patient portal is “an electronic application through which individuals can access, manage and share their health information in a secure and confidential environment. It allows people to access and coordinate their lifelong health information and make appropriate parts of it available to those who need it”.

**Do you have experience using a patient health record (PHR)?**

a) Yes

b) No

**Non Hypothetical version of the questionnaire**

**Using your PHR**

As a reminder, a patient health record (PHR) or patient portal is “an electronic application through which individuals can access, manage and share their health information in a secure and confidential environment. It allows people to access and coordinate their lifelong health information and make appropriate parts of it available to those who need it”. In this section, we wish to know how do you like your PHR to look like.

1. **What do you use your PHR for? (Check all that apply)**
   1. To access my health information
   2. To access my child(ren)’s health information
   3. To access the health information of someone whom I am caring for
   4. Other, please specify
2. **What do you like most about using your PHR? (Check all that apply)**
   1. It is easier to communicate with my family’s care providers.
   2. It is easier to schedule appointments for myself and my family.
   3. I like being able to see my own lab results.
   4. I like to be able to view my own health information.
   5. I like to be able to view health information for people I care for, such as my
   6. family members.
   7. I like to be able to fill out clinical questionnaires in advance of my appointment.
   8. I like to be able to record my Blood Pressure for my care providers.
   9. I like to be able to record my glucose levels for my care providers.
   10. Other, please specify
3. **Please indicate how much you agree with each of the following statements (Strongly disagree, Disagree, Neutral, Agree, Strongly agree)**
   1. PHR is easy to use
   2. I think PHR makes my care providers and clinic staff ask me to repeat myself

less often

- 1. PHR saves me time when scheduling an appointment
  2. It is more convenient to communicate with my care providers using PHR than using the method that I used to.

1. **Do you find your PHR helpful? (Please rate from 0 = Completely unhelpful to 100 = Completely helpful)**
2. **Please tell us how we could improve your PHR.**

|  |
| --- |

**Using your PHR**

As a reminder, a patient health record (PHR) or patient portal is “an electronic application through which individuals can access, manage and share their health information in a secure and confidential environment. It allows people to access and coordinate their lifelong health information and make appropriate parts of it available to those who need it”.

In this section we wish to know whether your PHR is helpful to you.

1. **Do you think that using your PHR has helped you/your family avoiding an Emergency Department (A&E) or Urgent Care visit?**
   1. No
   2. Yes (please explain how)

|  |
| --- |

1. **Do you think that using your PHR has helped you/your family avoid a clinic visit(s)? (Check all that apply)**
   1. No
   2. Yes, because lab test or imaging results were discussed by secure messaging my care team.
   3. Yes, because a question that I sent via PHR secure messaging was answered in a way that avoided a visit.
   4. Yes, because of another reason (please specify)
2. **If you think that using your PHR has helped you/your family avoid a clinic visit, Please indicate which of these costs you saved on (check all that apply).**
   1. Petrol
   2. Time off work
   3. Getting childcare
   4. Parking
   5. Taxi
   6. Other (please specify)
3. **Where do you access your PHR from? (Check all that apply)**
   1. Desktop computer/ Laptop
   2. Smart phone
   3. Tablet device
   4. No preference

**Your health**

Below are some statements that people sometimes make when they talk about their health. Please indicate how much you agree or disagree with each statement as it applies to you personally. Your answers should be what is true for you and not what you think others might want you to say.

1. **Please indicate how much you agree with each of the following statements. (Strongly disagree, Disagree, Neutral, Agree, Strongly agree)**
   1. When all is said and done, I am the person who is responsible for taking care of my health.
   2. Taking an active role in my own health care is the most important thing that affects my health.
   3. I am confident I can help prevent or reduce problems associated with my health.
   4. I know what each of my prescribed medications does.
   5. I am confident that I can tell whether I need to go to the doctor or whether I can take care of a health problem myself.
   6. I am confident that I can tell a doctor the concerns I have even when he or she does not ask.
   7. I am confident that I can follow through on medical treatments I may need to do at home.
   8. I understand my health problems and what causes them.
   9. I know what treatments are available for my health problems.
   10. I have been able to maintain (keep up with) lifestyle changes, like eating right or exercising.
   11. I know how to prevent problems with my health.
   12. I am confident I can figure out solutions when new problems arise with my health.
   13. I am confident that I can maintain lifestyle changes, like eating right and exercising, even during times of stress.

**Who are you?**

1. **What is your gender?**
   1. Female (including trans female)
   2. Male (Including trans male)
   3. Decline to answer
   4. Other (please specify)
2. **What is your age range (in years)?**
   1. <18
   2. 18-29
   3. 30-39
   4. 40-49
   5. 50-59
   6. 60-69
   7. 70 +
3. **What is your level of comfort with computers?**
   1. Completely uncomfortable
   2. Uncomfortable
   3. Neither comfortable nor uncomfortable (Neutral)
   4. Comfortable
   5. Completely comfortable
4. **Your PHR has the ability to connect your record to another person called a carer. Please describe who you have connected carer accounts to (check all that apply, or leave blank for none)**
   1. My partner
   2. My parent/s
   3. My child(ren)
   4. Other please describe.
5. **Are you responsible for the care of family members? (check all that apply or leave blank for none)**
   1. My partner
   2. My parent/s
   3. My child(ren)
   4. Other (please specify)

**Hypothetical version of the questionnaire**

**Using your PHR**

As a reminder, a patient health record (PHR) or patient portal is “an electronic application through which individuals can access, manage and share their health information in a secure and confidential environment. It allows people to access and coordinate their lifelong health information and make appropriate parts of it available to those who need it”.

In this section, we wish to know how do you like your PHR to look like.

1. **If you had a PHR, what do you imagine you would use it for? (Check all that apply)**
   1. To access my health information
   2. To access my child(ren)’s health information
   3. To access the health information of someone whom I am caring for
   4. Other (please specify)
2. **If you had a PHR, what do you imagine you would like most about using your PHR? (Check all that apply)**
   1. It would make it easier to communicate with my family’s care providers.
   2. It would make it easier to schedule appointments for myself and my family.
   3. I would like to be able to view my own lab results.
   4. I would like to be able to view my own health information.
   5. I would like to be able to view health information for people I care for, such as my family members.
   6. I would like to be able to fill out clinical questionnaires in advance of my appointment.
   7. I would like to be able to record my Blood Pressure for my care providers.
   8. I would like to be able to record my glucose levels for my care providers.
   9. Other (please specify)
3. **If you had a PHR, please indicate how much you imagine that you would agree with each of the following statements. (Strongly disagree, Disagree, Neutral, Agree, Strongly agree)**
   1. I imagine it would be easier to communicate with my care providers using the PHR rather than using the method that I am used to.
   2. I imagine that a PHR would make my care providers and clinic staff ask me to repeat myself less often.
   3. I imagine the PHR would save me time when I have to schedule an appointment.
4. **How helpful do you imagine that you would find your PHR?** (Please rate from 0 = Completely unhelpful to 100 = Completely helpful)

**Using your PHR**

As a reminder, a patient health record (PHR) or patient portal is “an electronic application through which individuals can access, manage and share their health information in a secure and confidential environment. It allows people to access and coordinate their lifelong health information and make appropriate parts of it available to those who need it”.

In this section we wish to know whether your PHR is potentially helpful to you.

1. **If you had a PHR, do you imagine that using your PHR would help you/your family avoid an emergency department (A&E) or urgent care visit(s)?**
   1. No
   2. Yes (please explain how)
2. **If you had a PHR, do you imagine that using your PHR would help you/your family avoid a clinic visit(s)?**
   1. No
   2. Yes
3. **If you had a PHR, please indicate which of these costs you imagine you would save on, by avoiding a clinic visit to your Doctor? (check all that apply).**
   1. Petrol
   2. Time off work
   3. Getting childcare
   4. Parking
   5. Taxi
   6. Other (please specify)
4. **If you had a PHR, where do you imagine you would access your PHR from? (Check all that apply)**
   1. Desktop computer/ Laptop
   2. Smart phone
   3. Tablet device
   4. No preference
5. **If you had a PHR, which do you imagine would be your preferred way of accessing your PHR?**
   1. Desktop computer/ Laptop
   2. Smart phone
   3. Tablet device
   4. No preference

**Your health**

Below are some statements that people sometimes make when they talk about their health. Please indicate how much you agree or disagree with each statement as it applies to you personally. Your answers should be what is true for you and not what you think others might want you to say.

1. **Please indicate how much you agree with each of the following statements. (Strongly disagree, Disagree, Neutral, Agree, Strongly agree)**
   1. When all is said and done, I am the person who is responsible for taking care of my health.
   2. Taking an active role in my own health care is the most important thing that affects my health.
   3. I am confident I can help prevent or reduce problems associated with my health.
   4. I know what each of my prescribed medications does.
   5. I am confident that I can tell whether I need to go to the doctor or whether I can take care of a health problem myself.
   6. I am confident that I can tell a doctor the concerns I have even when he or she does not ask.
   7. I am confident that I can follow through on medical treatments I may need to do at home.
   8. I understand my health problems and what causes them.
   9. I know what treatments are available for my health problems.
   10. I have been able to maintain (keep up with) lifestyle changes, like eating right or exercising.
   11. I know how to prevent problems with my health.
   12. I am confident I can figure out solutions when new problems arise with my health.
   13. I am confident that I can maintain lifestyle changes, like eating right and exercising, even during times of stress.

**Who are you?**

1. **What is your gender?**
   1. Female (including trans female)
   2. Male (Including trans male)
   3. Decline to answer
   4. Other (please specify)
2. **What is your age range (in years)?**
   1. <18
   2. 18-29
   3. 30-39
   4. 40-49
   5. 50-59
   6. 60-69
   7. 70 +
3. **What is your level of comfort with computers?**
   1. Completely uncomfortable
   2. Uncomfortable
   3. Neither comfortable nor uncomfortable (Neutral)
   4. Comfortable
   5. Completely comfortable
4. **Your PHR has the ability to connect your record to another person called a carer. If you had a PHR, please describe who you imagine you would connect carer accounts to (check all that apply or leave blank for none)**
   1. My partner
   2. My parent/s
   3. My child(ren)
   4. Other please describe.
5. **Are you responsible for the care of family members? (check all that apply or leave blank for none)**
   1. My partner
   2. My parent/s
   3. My child(ren)
   4. Other (please specify)
